# Bach Adsorption Study for the Extraction of Silver Ions by Hydrazone Compounds from Aqueous Solution

**DOI:** 10.1100/2012/351967

**Published:** 2012-04-26

**Authors:** Abdussalam Salhin Mohamad Ali, Norfarhah Abdul Razak, Ismail Ab Rahman

**Affiliations:** ^1^School of Chemical Sciences, Universiti Sains Malaysia, 11800 Penang, Malaysia; ^2^School of Dental Sciences, Universiti Sains Malaysia, Kelantan, 16150 Kubang Kerian, Malaysia

## Abstract

Sorbent materials based on a hydrazone Schiff base compound, C_14_H_11_BrN_4_O_4_, were prepared either by immobilizing the ligand into sol-gel (SG1) or bonding to silica (SG2). The sorbent materials were characterized by FT-IR, EDX, SEM, TEM, and TGA. The sorption characteristics of a matrix of eight transition metal ions (Ag^+^, Cu^2+^, Co^2+^, Ni^2+^, Fe^3+^, Pb^2+^, Zn^2+^, and Mn^2+^) using batch method were studied. Several key parameters that affected the extraction efficiency such as pH, contact time, metal ions concentration, and gel size (for SGl) were investigated and optimized. Under the optimized conditions, the physically immobilized hydrazone sorbent (SG1) exhibits highest selectivity towards Ag^+^ ions, while the chemically bonded hydrazone sorbent (SG2) exhibits high extraction for all metal ions tested. However, for practical applications such as the removal and preconcentration of Ag^+^, the physically immobilized sorbent (SG1) is preferred.

## 1. Introduction

Hydrazone compounds are one of the important classes in organic chemistry that have been widely studied nowadays. It drawn such of attention because of their biological and pharmaceutical activities [1], such as anticancer [2–4], anti-inflammatory [2], analgesic [2, 4], antipyretic [2], and antibacterial [2, 5]. These compounds also possessed antioxidative [3], antimalarial [4, 6], antitubercular [5–7], antiplatelet [4], antifungal [8, 9] activities and potential inhibitor for many enzymes [10–12], DNA synthesis, and cell growth [13] activities. Some of these compounds have been commercialized, for example, Nifurtimox used for the treatment of Chaga's disease [6] and Nifuroxazide (D) as intestinal antiseptic [5].

Hydrazone compounds can act as multidentate ligands depending on the nature of the substituent attached to the hydrazone unit [14]. They are also used as analytical reagents, polymer-coating, ink, pigments, and fluorescent materials [15]. They can form a very stable complexes with different metal ions giving well-characterized metal complexes [16, 17]. They form a coloured chelates with transition elements which are then used in the selective and sensitive determination of these metal ions [18]. Accordingly several hydrazone compounds were synthesized and their applications in the spectrophotometric determination of trace amounts of metal ions such as cobalt [19], calcium [20], lanthanides [21–25] and anions such as acetate [26, 27] were reported.

These compounds can be directly complex with metal ions or immobilize them in a matrix as a new class of materials for application in selected fields. To achieve this goal, two ways of immobilization following either processes, chemically or physically in sol-gel matrix, were applied. Sol-gel process is closely controlled by the initial synthesis conditions such as temperature, water, acid, or basic. Accordingly, different ranges of final products such as powders, monolithic gels, and thin films were produced. In the second type the ligands were covalently bonded to the silica backbone.

Silica gel incorporated Schiff base compounds such as 2,4-dinitrophenylhydrazine (used for the determination of airborne aldehydes and ketones in air samples) [28, 29], bis-(3-methoxysalicylaldehyde)-1,6-diaminohexane, and benzophenone 4-aminobenzoyl hydrazone (chelating collectors for metal ions) [30, 31] were reported. Recently [32, 33], our group reported the solid phase extraction of biogenic amines from food samples based on newly synthesized hydrazone compounds incorporated on sol-gel matrix. High extraction efficiency was achieved between the sol-gel sorbent that contained the hydrazone ligand and the target analyte. We have also reported the application of 1,4-bis(4-dimethylaminobenzyl)-2,3-diaza-1,3-butadiene hydrazone as colorimetric reagent for Cu^2+^ [34].

This paper investigates the extraction efficiency of a hydrazone ligand immobilized or bonded to silica towards metal ions. The optimum conditions for a better extraction such as pH, contact time, concentration of metal ions, sorbent particle size, foreign metal ions, mass, and reusability of the sorbent were investigated.

## 2. Experimental

### 2.1. Apparatus

FTIR spectra were recorded using a Perkin-Elmer Spectrum BX Fourier transform infrared spectrometer (FT-IR) system using KBr in the range 4000.0–400.0 cm^−1^. LEO Supra 50 vp field emission scanning electron microscope (SEM) equipped with Oxford INCA 400 energy dispersive X-ray microanalysis system (EDX) was used to study the surface morphology. The surface of the SG was visualized using a transmission electron microscope (TEM), Phillips CM12 with Docu version 3.2 image analysis. A Perkin-Elmer lambda 35 (dual beam) spectrophotometer equipped with a solid-state sample holder was used for UV-Vis solid sample analysis. A mechanical shaker (Stuart Scientific, UK) was used for extraction. Deionized water was produced from Millipore Milli-Q plus. Extractant concentrations were analyzed using a Perkin-Elmer AAnalyst 200 atomic absorption spectrometer (AAS). TGA was recorded on Perkin Elmer TGA-7 series thermal analyzer. 

### 2.2. Chemicals and Reagents

One hundred milliliters stock solution (2 ppm) for each metal ions (Ag^+^, Cu^2+^, Co^2+^, Zn^2+^, Pb^2+^, Fe^3+^, Ni^2+^, and Mn^2+^) were prepared from stock solution of metal ions (1000 ppm, Merck) in distilled water. Hydrazone compound, 1-[(bromomethyl)(phenyl)methyl]-2-(2,4-dinitrophenyl) hydrazine (BPMDNPH), was prepared using reported route [35]. Tetraethoxysilane (TEOS) (Fluka) was used as sol-gel precursor, and 3-aminopropyltriethoxysilane (Fluka) for silica modification, hydrochloric acid (R&M Chemicals), nitric acid (Systerm), ammonia (R&M Chemicals), ethanol (EtOH) (QRëC) and tetrahydrofuran (THF) (Fluka) were used as received. Silica gel (Aldrich) was used for chemically immobilized SG. For the determination of Ag^+^ in real sample, river water was collected from Waterfall River, Penang, Malaysia. The water was filtered to remove suspended particles. 

### 2.3. Preparation of Sol-Gel Immobilized BPMDNPH (SG1)

The sol solution was prepared by stirring a mixture of TEOS (6.56 mL), EtOH (9.12 mL), and HCl (1.0 M, 0.72 mL) for 15 min. A THF solution of BPMDNPH was added separately to the sol solution and stirred vigorously for 45 min. The resulting clear solution was aged in the oven (60°C) for 2 days. Shrinkage of the gel occurred causing it to crack during the drying stage. For conditioning, the prepared dried gel was then soaked in deionized water for 1 day and dried again at 60°C for another 1 day. The gel was ground into small pieces (1–5 mm diameter) using mortar and pestle. The same procedure was used for the preparation of the blank sorbent except that no hydrazone ligand was added.

### 2.4. Preparation of Silica-Gel Bonded BPMDNPH (SG2)

First, the commercial silica gel (60 Å, 35–70 mesh, Aldrich) was activated by refluxing in 1.0 M hydrochloric acid for 4 h. It was stored in a dry place after thorough washing with doubly distilled water until free from acid followed by drying for 12 h in an oven at (110°C). The activated silica (5.0 g) was suspended in 50 mL of dry toluene, and then an excess of 3-aminopropyltriethoxysilane (5.0 mL) was added. The mixture was refluxed with stirring for 24 h. After refluxing, the reaction was stopped and the modified silica was cooled to room temperature. The slurry was filtered and the resulting solid, namely, silica-gel-bond-3-aminopropyl phase (SGBAP), was washed successively with toluene, ethanol, and diethyl ether. SGBAP was dried under vacuum at 60°C for 8 h, before proceed to the second reaction.

**Figure 1:**
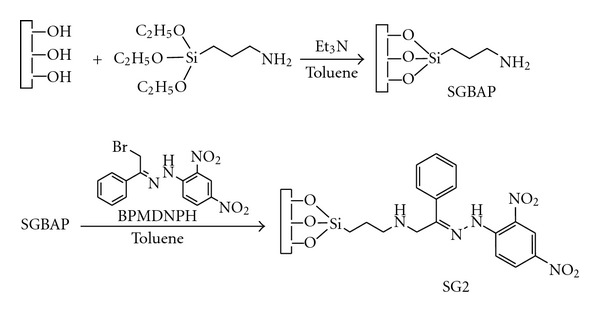
The schematic diagram of the synthetic approach for the preparation of SG2.

Secondly, the chemically bonded aminopropyl group on the silica surface (SGBAP) was reacted with BPMDNPH. In brief, 5.0 g of dry SGBAP was placed in a reaction flask containing 50 mL of dry toluene, 0.5 mL of triethylamine and an excess of BPMDNPH were used. The mixture was refluxed with stirring for 12 h. After refluxing, the reaction was stopped and the modified silica (SG2) was cooled to room temperature, transferred to a vacuum glass filter, and washed with toluene (100 mL), ethanol (100 mL), and diethylether (50 mL). The product (SG2) was dried under vacuum at 60°C for 6 h prior to packing or characterization.  Figure 1 shows the schematic diagram of the synthetic approach for the preparation of SG2 silica.

### 2.5. Extraction of Metal Ions—Batch Method

Batch method of extraction was conducted. The sorbent (0.1 g, SG1 or SG2) was placed in a glass vial along with 6 mL metal ions solution (2 ppm) and the pH of the solution was adjusted using nitric acid and ammonia solution. The mixture was shaken mechanically at room temperature (25°C) for 1 h. After the equilibrium time, the mixture was filtered and the amount of the unextracted metal ion (left in the solution after the extraction) was determined by AAS. The concentrations of the extracted metal ions were calculated by the difference. Once the extraction was completed, the metal ion was desorbed by shaking the sorbent with 1.0 M HNO_3_ (5 mL) for 30 min. The solution was filtered and the amount of the stripped metal ion was determined by AAS. The sorbent was rinsed several times with water and dried (60°C) before the next extraction cycle was conducted. The extraction and stripping cycle were repeated five times.

## 3. Results and Discussion

### 3.1. FT-IR Analysis

The spectrum of BPMDNPH hydrazone ligand shows a characteristic absorption band at 1620 cm^−1^ due to >C=N– stretching. The >NH and NO_2_ stretching bands, appeared at 3320 cm^−1^, and 1340 cm^−1^, respectively. Both blank sol-gel and BPMDNPH sol-gel (SG1) showed a similar spectrum. SG1 gel shows different intensity of peaks compared to the blank gel. The presence of a broad and intense absorption bands in both spectra between 1350 and 1000 cm^−1^ with maximum peak at approximately 1083 cm^−1^ is assigned to the Si–O–Si band. However, for SG1 spectra no >C=N– peak appears as observed in the free hydrazone spectrum. For the second material (SG2), a slight shift for both spectra was observed. The SG2 spectra show a stretching band at 3455 cm^−1^ which was due to the presence of >NH group. Shifting in bands was observed in comparison between the activated and modified (SG2) silica.

**Figure 2:**
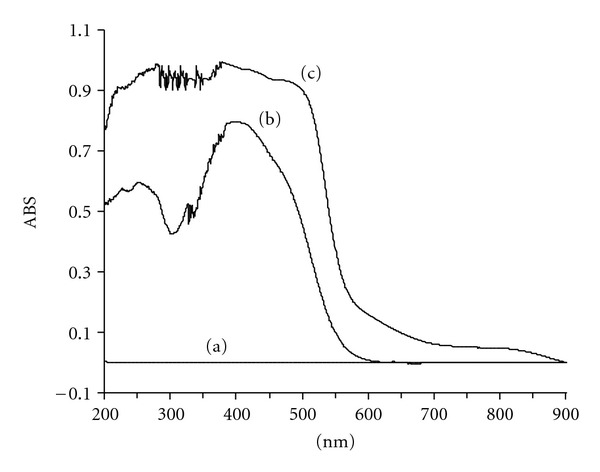
UV-Vis spectra of (a) blank gel, (b) SG1 sorbent, and (c) free ligand (BPMDNPH).

Further analysis on the ligand immobilization was conducted using solid-state UV-Vis technique.  Figure 2 shows a comparison between the free ligand (BPMDNPH) and its corresponding sorbent (SG1). The free ligand showed red shift with maximum absorption (*λ*_max⁡_) between 320–520 nm. This range is comparable with Uchiyama et al., [36] report on aldehyde-2,4-dinitrophenylhydrazone derivatives. There is no significant absorbance for the blank SG at this range. The gel material, SG1, exhibited similar phenomenon at *λ*_max⁡_ equals to 400 nm. The difference between the blank and immobilized gels is strong evidence that indicates the successful incorporation of BPMDNPH into the gel network. The clear shift in the wavelength of the ligand could be due to intermolecular interaction between the ligand and the silica, which causes the change in the characteristics of the spectrum [37].

### 3.2. EDX, SEM, and TEM Analysis

EDX analysis confirmed the immobilization of BPMDNPH in the silica matrix.  Table 1 presented the results of the blank SG and sol-gel immobilized BPMDNPH both physically (SG1) and chemically (SG2). For the blank SG, the main elements are Si (44.2%) and O (34.35%). This agrees well with the FTIR findings of the blank SG, in which the Si–O–Si is the backbone of this material. Since the hydrazone compound is absent in the blank SG, no nitrogen was detected. Further analysis on the immobilized gels confirmed the presence of nitrogen (1.28% & 1.90% for SG1 and SG2, resp.). Indicating the presence of the hydrazone ligand (BPMDNPH) in the SG matrix, most likely in physically (SG1) or chemically (SG2) bonded.

**Figure 3:**
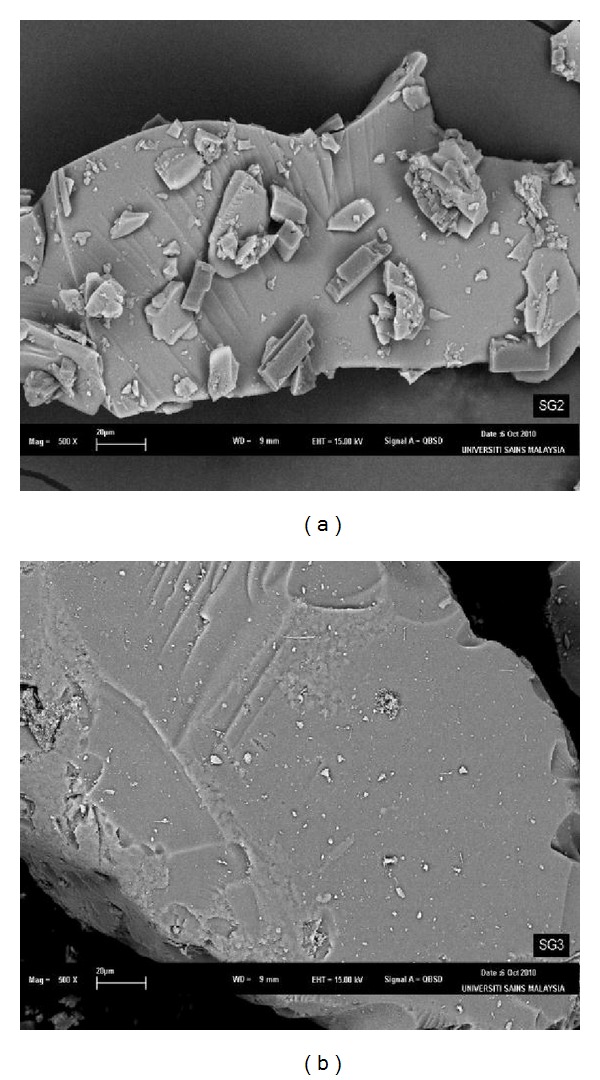
Scanning electron micrographs of (a) SG1 and (b) SG2.

**Figure 4:**
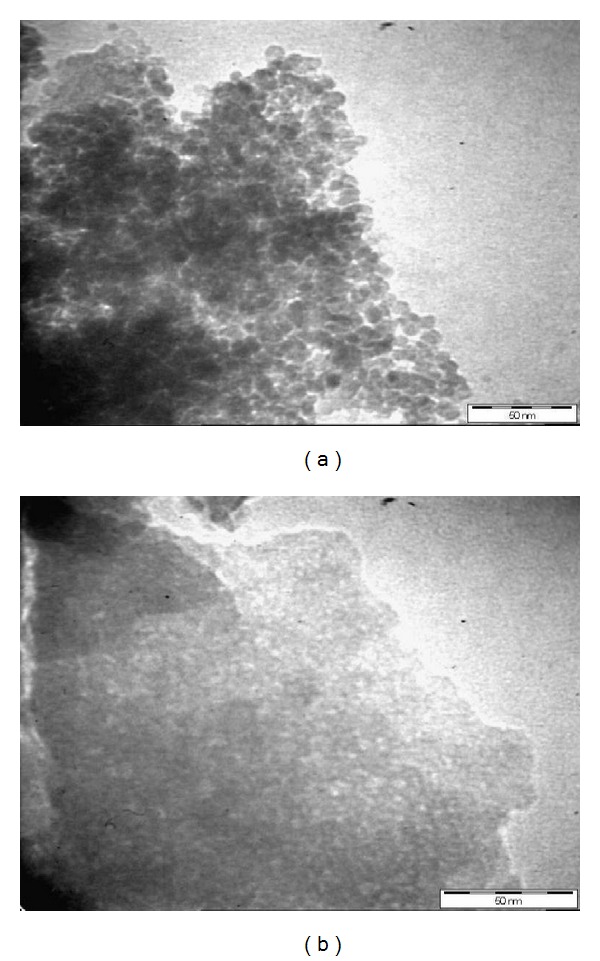
TEM of (a) BPMDNPH-SG1 and (b) BPMDNPH-SG2.

Figure 3 shows the surface of both SG1 and SG2 obtained from the SEM analysis. Smooth and homogenous surfaces were observed for both gels. It can be concluded that SG2 had smoother surface than SG1. Further analysis using transmission electron microscope (TEM) was conducted to visualize the surface of both silica adsorbents. Pores can be easily seen (Figure 4) as white and tiny dots with approximate diameter equals to 2.0–4.0 nm and 4.0–10.0 nm for both sorbents, respectively. This reflected the porosity and the distribution of ligand (BPMDNPH) particles on the sorbent materials.

### 3.3. Thermogravimetric Analysis

**Figure 5:**
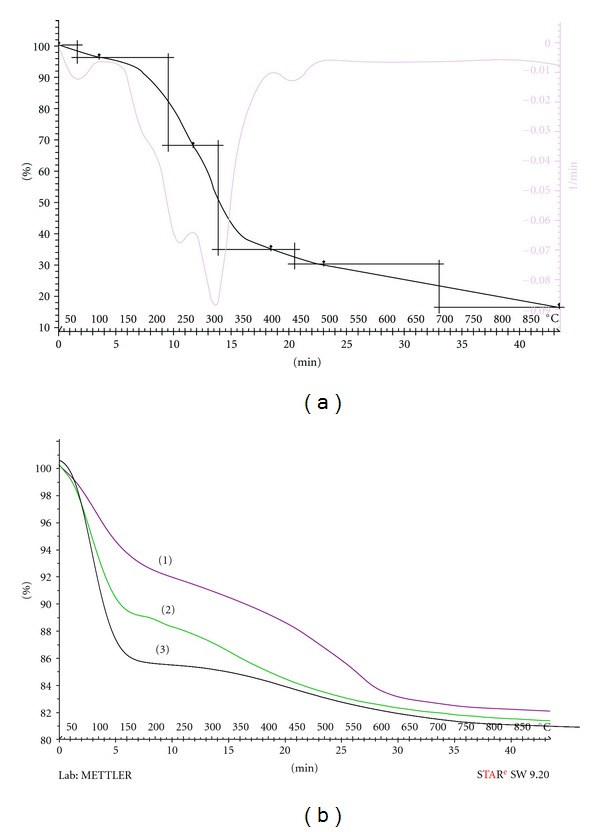
Thermal studies for decomposition process of (a) BPMDNPH, (b) SG1 (1), SG2 (2), and blank sol-gel (3).

Thermogravimetric analysis (TGA) was used to study the thermal stability of the silica materials over 30–900°C under nitrogen atmosphere at constant heating rate of 10°C per min. The results obtained from the analysis are shown in Figure 5. Temperature of the initial decomposition of the pure ligand was lower compared with the blank, SG1 and SG2 sorbents. The blanks gel decomposes in two distinct steps. The first loss of weight which takes place below 150°C is associated with the physical desorption of water. The second and abroad peak is probably due to the evaporation of trapped water molecules or due to the combustion of the organic components [38]. The sorbents produced similar thermal characteristics with the blank gel. Both sorbents lost <15% of their mass below 150°C that associated with the loss of physically absorbed water as the case for the blank gel. However, there is no significant loss in the range 240–300°C compared with the free ligand, indicating the enormous enhancement on the thermal stability of the ligand upon immobilization on silica matrices.

### 3.4. Adsorption Study

#### 3.4.1. Extraction of Metal Ions with SG1/SG2 (Batch Method)

**Effect of pH**

**Figure 6:**
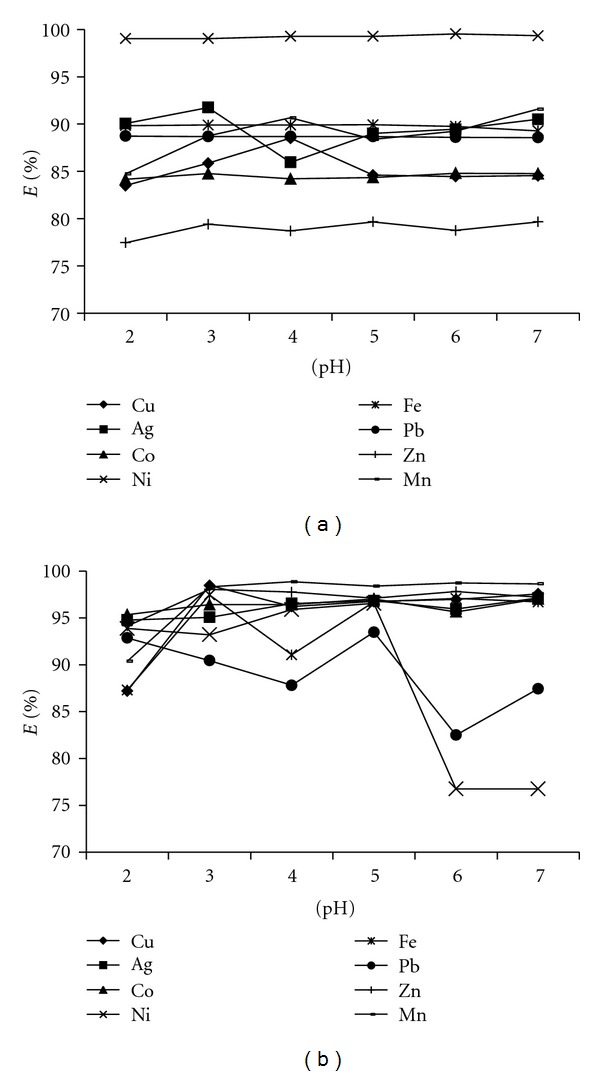
Effect of pH on the extraction of metal ions (2 ppm each) with 0.1 g of (a) SG1 and (b) SG2. Shaking time: 1 hour.

Among the chemical variables, pH was the most critical parameter affecting the formation and retention of the metal-BPMDNPH complex on the sorbent. In order to evaluate the effect of pH on the extraction efficiency, the pH of the sample solution containing a mixture of metal ions (2 ppm each) was adjusted in the range of 2–7. The metal ions were then extracted with 0.1 g sorbent material, SG1 and SG2 (separately). From our previous study [39], it was found that the blank gel had an efficiency to extract metal ion from slightly acidic medium (pH5). In the present study, almost all metal ions were highly extracted (~85%) at the studied pH range (Figure 6). The extraction efficiency of the sorbents at higher pH (>7) was not conducted because of the hydrolysis of studied metal ions. For SG1, silver was found the most extracted among the studied metal ions. The same procedure was applied for determination of the extraction efficiency of SG2. All metal ions were extracted constantly in high percentage (>85%) at the same pH range (Figure 6(b)). Less extraction was observed for Pb^2+^  and Ni^+^ at pH higher than 5. Since all metal ions were highly extracted at pH 5, this pH was chosen for further extraction using both sorbents.

**Effect of Contact Time**

**Figure 7:**
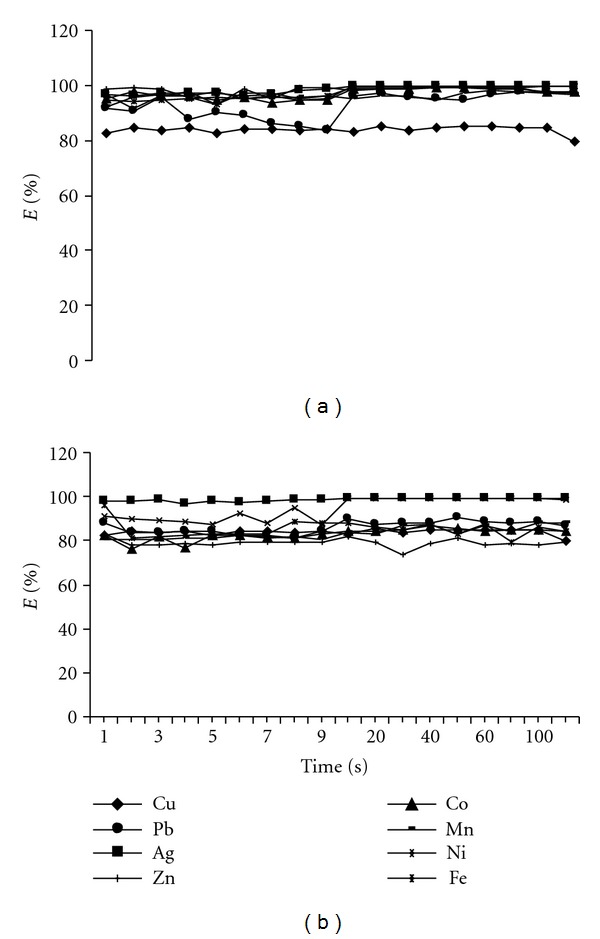
Effect of contact time on the extraction of metal ions with 0.1 g of (a) SG1 and (b) SG2 conducted using batch method, pH 5.

The extraction was carried out by shaking mechanically the sorbent matrices with the metal ions solution ranging from 1 to 120 min at their optimum pH 5 at room temperature. Results obtained were illustrated in Figure 7. Over 95% of Ag^+^ was extracted within the first minute of contact. Increasing the contact time has no significant effect on the extraction of Ag^+^ ions by both sorbents. This equilibrium time is better than our previous finding about the solid phase extraction of Ag^+^ with crown ethers using the same technique [39]. No selectivity among the metal ions was observed for SG2 sorbent. This result also shows that SG2 could be used as an excellent sorbent for the extraction of all the metal ions compared with SG1 sorbent that shows slight selectivity towards Ag^+^. Accordingly, from the above analysis (pH and contact time), it can be concluded that Ag^+^ was the most extracted metal ion from acidic medium (pH 5) with BPMDNPH immobilized using silica gel (SG1). Therefore, Ag^+^ was chosen for further studies.

**Effect of Sol-Gel Size (SG1)**

**Figure 8:**
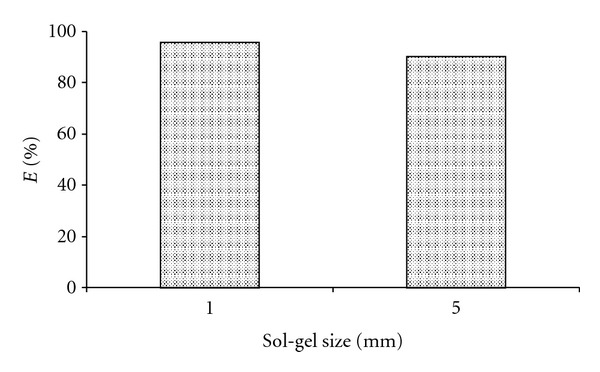
Effect of sol-gel size on the extraction (pH 5) of Ag^+^ with 0.1 g of SG1.

Two sizes (1 and 5 mm diameter) of gel (SG1) had been used to study the effect of the gel size on the extraction of Ag^+^ from aqueous medium (pH5). It was observed that the smaller size (1 mm) of SG1 exhibits higher extraction percentage (>96%) than 5 mm gel (90%) size (Figure 8). This may be due to larger surface area possess by smaller size of gel, which increases the extraction effectiveness. This factor was not applied for SG2 sorbent, because the sorbent had a homogeneous particle size.

**Effect of Sorbent Mass**

**Figure 9:**
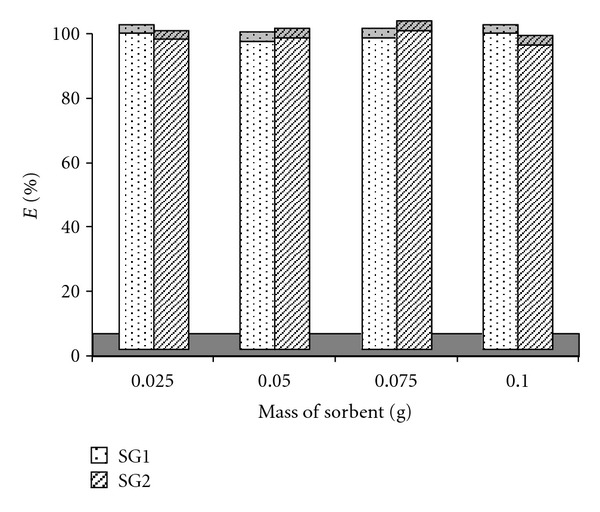
Effect of sorbent mass on the extraction of Ag^+^ with SG1 and SG2 sorbents, pH 5.

With the same size of dry gel (SG1), different amounts of SG1 were used to study the mass needed for effective extraction of metal ion. Accordingly, 0.025 to 0.100 g of SG1 was used. Results showed that the entire mass could be used to extract more than 95% of Ag^+^. Therefore, 0.1 g SG1 sorbent was used for further studies. The same mass range was also used for the SG2 sorbent. 0.075 g sorbent was the best amount of sorbent could be used to obtain the optimum extraction of Ag^+^ (~100%). Results for both sorbents are shown in Figure 9.

**Effect of Ag^+^Concentration**

The study of silver concentration gives the idea about the extraction capacity of the sorbent material. Therefore, the effect of Ag^+^ concentration on the extraction efficiency of the sorbents was also tested. Different concentration of Ag^+^  (0.01–100 ppm) was used for the extraction using both sorbent materials. All Ag^+^ were successfully extracted (>95%) by both sorbents (Table 2). Under the optimum conditions, the exchange process between the two phases (solid/liquid) can be characterized by the number of milligrams of Ag^+^ ion adsorbed (*Q*, mg L^−1^) per gram of sorbent [40, 41]. This value was calculated using the following equation:

(1) $$\begin{array}{c} Q=\;\frac{\left(C_{0}-C\right)V}{m} \end{array},$$

where *C*_0_ and *C* are the initial and equilibrium metal ion concentration (mg L^−1^) in solution, respectively. *V* is the volume of the solution (mL) and *m* is the mass of the sorbent material in grams. The amount (mg) of Ag^+^ sorbed per gram of sorbent, *Q*, is presented in Table 2. Thus, as can be seen, the sorbents capacity increased with the increase in initial Ag^+^ ion concentration. The capacity of each sorbent material for trace amount of silver was found to be up to 5.8 mg g^−1^ and 5.9 mg g^−1^. This shows that both sorbent materials exhibited similar capacity towards silver. Therefore, both sorbents (SG1 and SG2) are good extracting materials for trace amounts of Ag^+^ in aqueous comparing to other similar reported sorbents [39].

**Effect of Foreign Metal Ions**

Since SG1 shows higher capacity as well as better selectivity towards Ag^+^, the effect of other metal ions on the extraction of Ag^+^ by SG1 was investigated. The selective separation of Ag^+^ from binary mixture with Cu^2+^, Co^2+^, Zn^2+^, Pb^2+^, Fe^3+^, Ni^2+^, and Mn^2+^  was studied. A 2.0 ppm from the above-mentioned metal ions were mixed with an equivalent amount of Ag^+^ and the mixture was extracted with the SG sorbent at the optimum condition for Ag^+^. Quantitatively extraction of Ag^+^  was obtained (Table 3). A slight decrease in the extraction was observed compared to the value obtained in the absence of foreign metal ions. Based on the amount of metal ion remaining in the eluent, the distribution coefficient (*K*_*d*_) and separation factors (*K*) for Ag^+^ over other metal ions were calculated using the following equations [39]:

(2) $$\begin{array}{c} \begin{array}{c} K_{d}=\frac{C_{0}-C}{C} \end{array}\cdot \frac{V}{m},\\ K=\frac{K_{d({\text{Ag}}^{+})}}{K_{d({\text{M}}^{\text{n}+})}}, \end{array}$$

where M^n+^ = Cu^2+^, Ni^2+^, Fe^3+^, Pb^2+^, Zn^2+^, and Mn^2+^.

Table 3 showed that even in the presence of other metal ions, the sorbent material still shows better selectivity towards Ag^+^. This adsorbing selectivity corresponds to the sorbent high capacity and rate for Ag^+^ and could be attributed to the hard and soft acid and base theory [42]. Nitrogen atoms in the gel matrix have an easily polarizable ion pairs of electrons (soft base), which could interact strongly with soft acids such as Ag^+^ [43].

**Reusability of the SG1 Sorbent**

The performance of the sorbent material under repeated use was tested by performing the extraction-desorption three extraction cycles. The extraction efficiency of the sorbent material towards Ag^+^ is still high. Under the optimized conditions, Ag^+^ uptake was found always higher than the uptake of other metal ions at pH 5. Approximately, more than 98% of Ag^+^ could be achieved.

#### 3.4.2. Application to Environmental Water Samples

In order to validate the proposed method, the performances of the sorbent materials were tested on water samples. Two water samples, namely, tap water and drain water, were collected from the Universiti Sains Malaysia, Penang Campus, Malaysia. The water samples were filtered to remove suspended particles. The samples were first directly analyzed with AAS to determine their Ag^+^ contents. Ag^+^ was not detected in all samples. Therefore, all samples were spiked with 2.0 ppm of Ag^+^. Ag^+^ was successfully recovered (>94%) from both samples (Table 4). This low recovery may be due to the interference of other ionic species that would bond with the reactive sites of the ligand. The sorption characteristics of the studied sorbents are comparable with other SPE systems for the extraction/preconcentration of Ag^+^ ions that were previously published [39].

## 4. Conclusion

A novel compound, namely, 1-[(bromomethyl)(phenyl)methyl]-2-(2,4-dinitrophenyl) hydrazine (BPMDNPH) had been successfully immobilised (gel) or bonded on silica. These sorbents were characterized and used for extraction of metal ions. Several parameters for metal ions extraction with these sorbents had been optimized. Physically immobilized ligand (SG1) shows high selectivity towards Ag^+^ under optimized condition compared to the chemically bonded ligand (SG2). The results show that the gel sorbent (SG1) is capable to extract all the metal ions, with Ag^+^ as the best extracted. This sorbent can be used repeatedly three times without any significant deterioration. The developed method is successfully employed for analysis of water samples after successful validation. This added an advantage for the sorbent to be applied for several samples to extract Ag^+^  ions.

## Figures and Tables

**Table 1:** EDX results of blank sol-gel, SG1 and SG2.

Element	Material		
	Blank sol-gel	SG1	SG2
Carbon	21.45	23.49	14.17
Oxygen	34.35	33.80	43.96
Silicon	44.20	38.67	38.95
Nitrogen	—	1.28	1.90

**Table 2:** Effect of Ag^+^ concentration on the extraction efficiency of SG1 sorbent, pH 5, *n* = 5.

Ag^+^ (mg L^−1^)	SG1		SG2	
	%E	*Q* (mg g^−1^)	%E	*Q* (mg g^−1^)
0.01	90.00	0.0006	97.30	0.0006
0.05	96.00	0.0030	97.57	0.0029
0.1	99.00	0.0059	98.00	0.0059
0.2	99.50	0.0119	98.25	0.0118
0.5	98.40	0.0295	98.60	0.0296
1.0	98.00	0.0588	99.40	0.0596
1.5	96.00	0.0864	99.47	0.0895
2.0	98.30	0.1176	99.80	0.1198
10.0	98.32	0.5899	99.16	0.5950
20.0	98.95	1.1874	99.50	1.1940
50.0	99.07	2.9721	99.67	2.9901
100.0	96.46	5.7876	98.42	5.9052

**Table 3:** Effect of foreign metal ions (2 ppm) on the extraction of Ag^+^ (2 ppm) with SG1. pH 5, *n* = 4.

Mixture	%E (±SD)	*K*_*d*_	*K*
Ag^+^	99.27 (0.33)	8159.18	
Ag^+^+ Cu^2+^	94.30 (0.51)	992.63	28.55
Ag^+^+ Co^2+^	96.15 (0.61)	1498.44	28.75
Ag^+^+ Ni^2+^	94.75 (0.55)	1082.86	19.43
Ag^+^+ Fe^3+^	95.10 (0.65)	1164.49	27.41
Ag^+^+ Pb^2+^	94.85 (0.59)	1105.05	25.18
Ag^+^+ Zn^2+^	96.30 (0.57)	1583.31	36.91
Ag^+^+ Mn^2+^	93.90 (0.53)	923.61	30.87

**Table 4:** Determination of Ag^+^ in different water samples (spiked with 2 ppm Ag^+^).

Water sample	SG1		SG2	
	Found (ppm)	Recovered (%)	Found (ppm)	Recovered (%)
Tape	1.88	94.00	1.97	98.50
Drain	1.89	94.50	1.99	99.50

## References

[1] Sondhi SM, Dinodia M, Kumar A (2006). Synthesis, anti-inflammatory and analgesic activity evaluation of some amidine and hydrazone derivatives. *Bioorganic and Medicinal Chemistry*.

[2] Cocco MT, Congiu C, Lilliu V, Onnis V (2006). Synthesis and in vitro antitumoral activity of new hydrazinopyrimidine-5-carbonitrile derivatives. *Bioorganic and Medicinal Chemistry*.

[3] Wang BD, Yang ZY, Crewdson P, Wang DQ (2007). Synthesis, crystal structure and DNA-binding studies of the Ln(III) complex with 6-hydroxychromone-3-carbaldehyde benzoyl hydrazone. *Journal of Inorganic Biochemistry*.

[4] Xia Y, Fan CD, Zhao BX, Zhao J, Shin DS, Miao JY (2008). Synthesis and structure-activity relationships of novel 1-arylmethyl-3-aryl-1H-pyrazole-5-carbohydrazide hydrazone derivatives as potential agents against A549 lung cancer cells. *European Journal of Medicinal Chemistry*.

[5] Küçükgüzel SG, Mazi A, Sahin F, Öztürk S, Stables J (2003). Synthesis and biological activities of diflunisal hydrazide-hydrazones. *European Journal of Medicinal Chemistry*.

[6] Melnyk P, Leroux V, Sergheraert C, Grellier P (2006). Design, synthesis and in vitro antimalarial activity of an acylhydrazone library. *Bioorganic and Medicinal Chemistry Letters*.

[7] Kaymakçioğlu BK, Rollas S (2002). Synthesis, characterization and evaluation of antituberculosis activity of some hydrazones. *Farmaco*.

[8] Loncle C, Brunel JM, Vidal N, Dherbomez M, Letourneux Y (2004). Synthesis and antifungal activity of cholesterol-hydrazone derivatives. *European Journal of Medicinal Chemistry*.

[9] Pandeya SN, Sriram D, Nath G, Declercq E (1999). Synthesis, antibacterial, antifungal and anti-HIV activities of Schiff and Mannich bases derived from isatin derivatives and N-[4-(4’-chlorophenyl)thiazol-2-yl] thiosemicarbazide. *European Journal of Pharmaceutical Sciences*.

[10] Xu GC, Zhang L, Liu L, Liu GF, Jia DZ (2008). Syntheses, characterization and crystal structures of mixed-ligand Cu(II), Ni(II) and Mn(II) complexes of N-(1-phenyl-3-methyl-4-propenylidene-5-pyrazolone)-salicylidene hydrazide containing ethanol or pyridine as a co-ligand. *Polyhedron*.

[11] Hassanien MM, Gabr IM, Abdel-Rhman MH, El-Asmy AA (2008). Synthesis and structural investigation of mono- and polynuclear copper complexes of 4-ethyl-1-(pyridin-2-yl) thiosemicarbazide. *Spectrochimica Acta A*.

[12] Singh PK, Kumar DN (2006). Spectral studies on cobalt(II), nickel(II) and copper(II) complexes of naphthaldehyde substituted aroylhydrazones. *Spectrochimica Acta A*.

[13] Sreeja PB, Kurup MRP, Kishore A, Jasmin C (2004). Spectral characterization, X-ray structure and biological investigations of copper(II) ternary complexes of 2-hydroxyacetophenone 4-hydroxybenzoic acid hydrazone and heterocyclic bases. *Polyhedron*.

[14] Kuriakose M, Prathapachandra Kurup MR, Suresh E (2007). Synthesis, spectroscopic studies and crystal structures of two new vanadium complexes of 2-benzoylpyridine containing hydrazone ligands. *Polyhedron*.

[15] El-Tabl AS, El-Saied FA, Plass W, Al-Hakimi AN (2008). Synthesis, spectroscopic characterization and biological activity of the metal complexes of the Schiff base derived from phenylaminoacetohydrazide and dibenzoylmethane. *Spectrochimica Acta A*.

[16] El-Behery M, El-Twigry H (2007). Synthesis, magnetic, spectral, and antimicrobial studies of Cu(II), Ni(II) Co(II), Fe(III), and UO_2_(II) complexes of a new Schiff base hydrazone derived from 7-chloro-4-hydrazinoquinoline. *Spectrochimica Acta A*.

[17] Bessy Raj BN, Kurup MRP (2007). N-2-Hydroxy-4-methoxyacetophenone-N′-4-nitrobenzoyl hydrazine: synthesis and structural characterization. *Spectrochimica Acta A*.

[18] Vasilikiotis GS, Stratis J (1975). Phenylhydrazones of pyridine-2-aldehyde and pyridine-4-aldehyde as new acid-base indicators. *Analytica Chimica Acta*.

[19] Park CI, Cha KW (1998). Spectrophotometric determination of trace amounts of cobalt with 2-hydroxybenzaldehyde-5-nitro-pyridylhydrazone in presence of surfactant after separation with Amberlite IRC-718 resin. *Talanta*.

[20] Silva M, Valcárcel M (1980). Spectrophotometric determination of microgram amounts of calcium in waters and foods using diphenylglyoxal bis(2-hydroxybenzoyl hydrazone). *The Analyst*.

[21] Ganjali MR, Matloobi P, Ghorbani M, Norouzi P, Salavati-Niasari M (2005). La(III) selective membrane sensor based on a new N-N Schiff’s base. *Bulletin of the Korean Chemical Society*.

[22] Ganjali MR, Norouzi P, Daftari A, Faridbod F, Salavati-Niasari M (2007). Fabrication of a highly selective Eu(III) membrane sensor based on a new S-N hexadentates Schiff's base. *Sensors and Actuators B*.

[23] Ganjali MR, Mirnaghi FS, Norouzi P, Adib M (2006). Novel Pr(III)-selective membrane sensor based on a new hydrazide derivative. *Sensors and Actuators B*.

[24] Zamani HA, Ganjali MR, Norouzi P, Adib M, Aceedy M (2006). Synthesis of N′-(1-pyridin-2-ylmethylene)-2-furohydrazide and its application in construction of a highly selective PVC-based membrane sensor for La(III) ions. *Analytical Sciences*.

[25] Zamani HA, Ganjali MR, Adib M (2007). Construction of a highly selective PVC-based membrane sensor for Ce(III) ions. *Sensors and Actuators B*.

[26] Shao J, Lin H, Yu M, Cai Z, Lin H (2008). Study on acetate ion recognition and sensing in aqueous media using a novel and simple colorimetric sensor and its analytical application. *Talanta*.

[27] Gupta VK, Goyal RN, Sharma RA (2008). Anion recognition using newly synthesized hydrogen bonding disubstituted phenylhydrazone-based receptors: Poly(vinyl chloride)-based sensor for acetate. *Talanta*.

[28] Vogel M, Pötter W, Karst U (2000). Characterization of a chemical artifact in the liquid chromatographic determination of 3-butyn-2-one using the 2,4-dinitrophenylhydrazine method. *Journal of Chromatography A*.

[29] Uchiyama S, Matsushima E, Tokunaga H, Otsubo Y, Ando M (2006). Determination of orthophthalaldehyde in air using 2,4-dinitrophenylhydrazine-impregnated silica cartridge and high-performance liquid chromatography. *Journal of Chromatography A*.

[30] Mashhadizadeh MH, Pesteh M, Talakesh M, Sheikhshoaie I, Ardakani MM, Karimi MA (2008). Solid phase extraction of copper (II) by sorption on octadecyl silica membrane disk modified with a new Schiff base and determination with atomic absorption spectrometry. *Spectrochimica Acta B*.

[31] Gübbük IH, Güp R, Ersöz M (2008). Synthesis, characterization, and sorption properties of silica gel-immobilized Schiff base derivative. *Journal of Colloid and Interface Science*.

[32] Basheer C, Wong W, Makahleh A (2011). Hydrazone-based ligands for micro-solid phase extraction-high performance liquid chromatographic determination of biogenic amines in orange juice. *Journal of Chromatography A*.

[33] Tameem AA, Saad B, Makahleh A, Salhin A, Saleh MI (2010). A 4-hydroxy-N’-[(E)-(2-hydroxyphenyl)methylidene]benzohydrazide-based sorbent material for the extraction-HPLC determination of biogenic amines in food samples. *Talanta*.

[34] Shalash M, Salhin A, Theng TT, Saleh MI, Saad B (2011). Spectroscopic studies of 1,4-bis(4-dimethylaminobenzyl)-2,3-diaza-1,3-butadiene as colorimetric reagent for Cu^2+^. *World Applied Sciences Journal*.

[35] Salhin A, Abdul Razak N, Rahman IA (2009). 1-[(Bromo-meth-yl)(phen-yl)meth-ylene]-2-(2,4-dinitro-phen-yl)hydrazine. *Acta Crystallographica Section E*.

[36] Uchiyama S, Ando M, Aoyagi S (2003). Isomerization of aldehyde-2,4-dinitrophenylhydrazone derivatives and validation of high-performance liquid chromatographic analysis. *Journal of Chromatography A*.

[37] Ramanathan K, Kamalasanan MN, Malhotra BD, Pradhan DR, Chandra S (1997). Immobilization and characterization of lactate dehydrogenase on TEOS derived Sol-Gel films. *Journal of Sol-Gel Science and Technology*.

[38] Chakrabarti K, Kim SM, Oh EO, Whang CM (2002). Thermal analysis of poly(dimethylsiloxane)-modified silica xerogels. *Materials Letters*.

[39] Saad B, Chong CC, Ali ASM (2006). Selective removal of heavy metal ions using sol-gel immobilized and SPE-coated thiacrown ethers. *Analytica Chimica Acta*.

[40] Li C, Pan J, Zou X, Gao J, Xie J, Yongsheng Y (2011). Synthesis and applications of novel attapulgite-supported Co(II)-imprinted polymers for selective solid-phase extraction of cobalt(II) from aqueous solutions. *International Journal of Environmental Analytical Chemistry*.

[41] Sadeghi S, Sheikhzadeh E (2009). Solid phase extraction using silica gel modified with murexide for preconcentration of uranium (VI) ions from water samples. *Journal of Hazardous Materials*.

[42] Myasoedova GV, Antokol’skaya II, Savvin SB (1985). New chelating sorbents for noble metals. *Talanta*.

[43] Abd El-Ghaffar MA, Abdel-Wahab ZH, Elwakeel KZ (2009). Extraction and separation studies of silver(I) and copper(II) from their aqueous solution using chemically modified melamine resins. *Hydrometallurgy*.

